# Engineering *Streptomyces albulus* to enhance ε-poly-L-lysine production by introducing a polyphosphate kinase-mediated ATP regeneration system

**DOI:** 10.1186/s12934-023-02057-7

**Published:** 2023-03-14

**Authors:** Hao Yang, Daojun Zhu, Lang Kai, Liang Wang, Hongjian Zhang, Jianhua Zhang, Xusheng Chen

**Affiliations:** Key Laboratory of Industrial Biotechnology, Ministry of Education, School of Biotechnology, Jiangnan University, 1800 Lihu Road, Wuxi, 214122 Jiangsu China

**Keywords:** Polyphosphate kinase, *Streptomyces albulus*, ε-Poly-L-lysine, ATP regeneration system, Polyphosphate

## Abstract

**Background:**

ε-Poly-L-lysine (ε-PL) is a natural and safe food preservative that is mainly produced by filamentous and aerobic bacteria *Streptomyces albulus*. During ε-PL biosynthesis, a large amount of ATP is used for the polymerization of L-lysine. A shortage of intracellular ATP is one of the major factors limiting the increase in ε-PL production. In previous studies, researchers have mainly tried to increase the oxygen supply to enhance intracellular ATP levels to improve ε-PL production, which can be achieved through the use of two-stage dissolved oxygen control, oxygen carriers, heterologous expression of hemoglobin, and supplementation with auxiliary energy substrates. However, the enhancement of the intracellular ATP supply by constructing an ATP regeneration system has not yet been considered.

**Results:**

In this study, a polyphosphate kinase (PPK)-mediated ATP regeneration system was developed and introduced into *S. albulus* to successfully improve ε-PL production. First, polyP:AMP phosphotransferase (PAP) from *Acinetobacter johnsonii* was selected for catalyzing the conversion of AMP into ADP through an in vivo test. Moreover, three PPKs from different microbes were compared by in vitro and in vivo studies with respect to catalytic activity and polyphosphate (polyP) preference, and PPK2B^cg^ from *Corynebacterium glutamicum* was used for catalyzing the conversion of ADP into ATP. As a result, a recombinant strain PL05 carrying coexpressed *pap* and *ppk2B*^*cg*^ for catalyzing the conversion of AMP into ATP was constructed. ε-PL production of 2.34 g/L was achieved in shake-flask fermentation, which was an increase of 21.24% compared with *S. albulus* WG608; intracellular ATP was also increased by 71.56%. In addition, we attempted to develop a dynamic ATP regulation route, but the result was not as expected. Finally, the conditions of polyP_6_ addition were optimized in batch and fed-batch fermentations, and the maximum ε-PL production of strain PL05 in a 5-L fermenter was 59.25 g/L by fed-batch fermentation, which is the highest ε-PL production reported in genetically engineered strains.

**Conclusions:**

In this study, we proposed and developed a PPK-mediated ATP regeneration system in *S. albulus* for the first time and significantly enhanced ε-PL production. The study provides an efficient approach to improve the production of not only ε-PL but also other ATP-driven metabolites.

**Supplementary Information:**

The online version contains supplementary material available at 10.1186/s12934-023-02057-7.

## Introduction

ε-Poly-L-lysine (ε-PL), one of the eight natural homopoly(amino acid), is generally composed of 25–35 L-lysine residues linked by ε-amino and α-carboxyl groups with a molecular weight range from 3200 to 4500 Da [1, 2]. ε-PL has excellent antibacterial activity with a broad spectrum, including activity against gram-positive and gram-negative bacteria, yeast and fungi, which is mainly attributed to its isopeptide bond and multiple amino groups [3]. In addition, this biopolymer is biodegradable, water-soluble, thermally stable and nontoxic to humans and the environment. As a result, ε-PL was first approved as a natural food preservative and designated as being generally recognized as safe (GRAS) by the US Food and Drug Administration. Currently, it has become one of the three major natural food preservatives derived from microbes in the world and has been used in Japan, South Korea, the United States and China [4]. In addition, ε-PL can be used in weight loss and health care products, as well as for drug carriers, gene carriers, biochips, bioelectronic coating agents, and novel water-absorbent materials [4, 5]. Thus, ε-PL is a promising biological product in fields related to food, cosmetics, medicine and biomaterials.ε-PL can be chemically synthesized by solid-phase synthesis and ring-opening polymerization [3, 6]. However, both methods often pose additional problems, such as complex reactions, low ε-PL yield, and toxicity [7]. Microbial production is a more attractive method for industrialization than chemical synthesis because it is more practical, efficient, economic and environmentally friendly. Many microorganisms, such as *Streptomyces* [8], *Kitasatospora* [9]*, Epichloë* [10]*, Bacillus* [11] and *Corynebacterium* [12], have been reported to produce ε-PL in nature, and the strain *S. albulus* [7] is a common ε-PL-producing strain. However, ε-PL production by these wild-type strains is extremely low (usually less than 0.2 g/L), which makes it difficult to meet the requirements of industrial production. To improve the ability of these producing strains to synthesize ε-PL, mutagenesis combined with resistance screening, genome shuffling, and ribosome engineering have been widely performed in recent decades [13]. Moreover, genetic engineering techniques to overexpress *vgb* (encoding hemoglobin) [14, 15], *amtB* (encoding ammonium transporter) [16], *pls* (encoding ε-PL synthetase) [17, 18], and *dapA* (encoding dihydrodipicolinate synthase) [19] have been adopted recently. As a result, the ε-PL production of those strains was enhanced dramatically, up to 10- or 20-fold higher than that of the wild-type strain. Many innovative fermentation strategies, such as two-stage pH control, pH shock, mixed carbon source fermentation, solid-state fermentation, in situ product removal fermentation, and immobilized cell fermentation, have been developed to increase the production of ε-PL [13]. Based on strain modification and fermentation process optimization, the highest ε-PL production achieved was 70.3 g/L, which exceeds the yield of most secondary metabolites produced by *Streptomyces* [20]. However, the production of ε-PL still urgently needs to be increased because its cost is still a limitation in the food industry.

A high oxygen demand is the most significant factor related to microbial production of ε-PL. This demand is caused by each isopeptide bond formed in the molecule of ɛ-PL consuming one molecule of ATP, which means that large amounts of ATP are necessary for ɛ-PL biosynthesis. Unfortunately, oxygen transfer efficiency is low during fermentation because the culture broth becomes viscous due to the filamentous nature, intertwined hyphae and high cell density of ɛ-PL-producing strains. To overcome oxygen shortage in the process of ε-PL production, Bankar and Singhal [21] proposed a two-stage dissolved oxygen control strategy to increase the biomass and ε-PL production of *S. noursei* NRRL 5126 through aeration and agitation. However, this method requires a higher stirring speed and aeration rate, which produces a large shear force on mycelium during growth, is unfavorable to ε-PL production and requires a large energy input. Xu et al. [22] developed a strategy to improve the oxygen supply for *S. albulus* PD-1 by oxygen carrier addition. They first tested the effect of several oxygen carriers on ε-PL production and found that n-dodecane (0.5%, v/v) added to the fermentation broth at the beginning of fermentation could efficiently maintain dissolved oxygen levels with > 32% saturation during fed-batch fermentation. Finally, a maximum ε-PL production of 30.8 g/L and a dry cell weight (DCW) of 33.8 g/L were achieved, which were enhanced by 31.6% and 20.7%, respectively, compared with oxygen-limited fermentation. Furthermore, the authors engineered *S. albulus* PD-1 by introducing the *Vitreoscilla* hemoglobin gene into its chromosome to elevate the strain’s oxygen uptake capacity [23]. With this change, the recombinant strain produced 34.2 g/L ε-PL, which is more than 50.7% higher than the production of the wild-type strain. These studies have addressed the oxygen limitation and enhanced ε-PL production, but they only relieved the shortage of intracellular ATP in ε-PL-producing strains from the perspective of oxygen supply.

There are many strategies to modify the ATP supply, including manipulating metabolic pathways, regulating ATP synthase and the electron transport chain and increasing the ADP supply [24]. Recently, the ATP regeneration system mediated by PPK, which uses inexpensive polyP as a phosphate donor, has attracted our attention [25, 26]. Lv et al. [27] introduced a PPK-mediated ATP regeneration system in *Corynebacterium glutamicum* to enhance L-glutamine production, resulting in a 22.9% increase in intracellular ATP. Krauser et al. [28] developed an ATP regeneration system with co-overexpressing *adk* (encoding adenosylate kinase) and *ppk* (encoding PPK) in permeabilized *E. coli* for biocatalytic synthesis of flaviolin during multistep synthesis of oxytetracycline. With this ATP regeneration system, the biocatalytic efficiency of ATP turnover was increased by 90-fold. The above studies demonstrate that the use of a PPK-mediated ATP regeneration system for improving intracellular ATP supply in the ɛ-PL-producing strain is feasible. However, there is no report on enhancing ɛ-PL production through strengthening ATP supply in vivo.

**Fig. 1:**
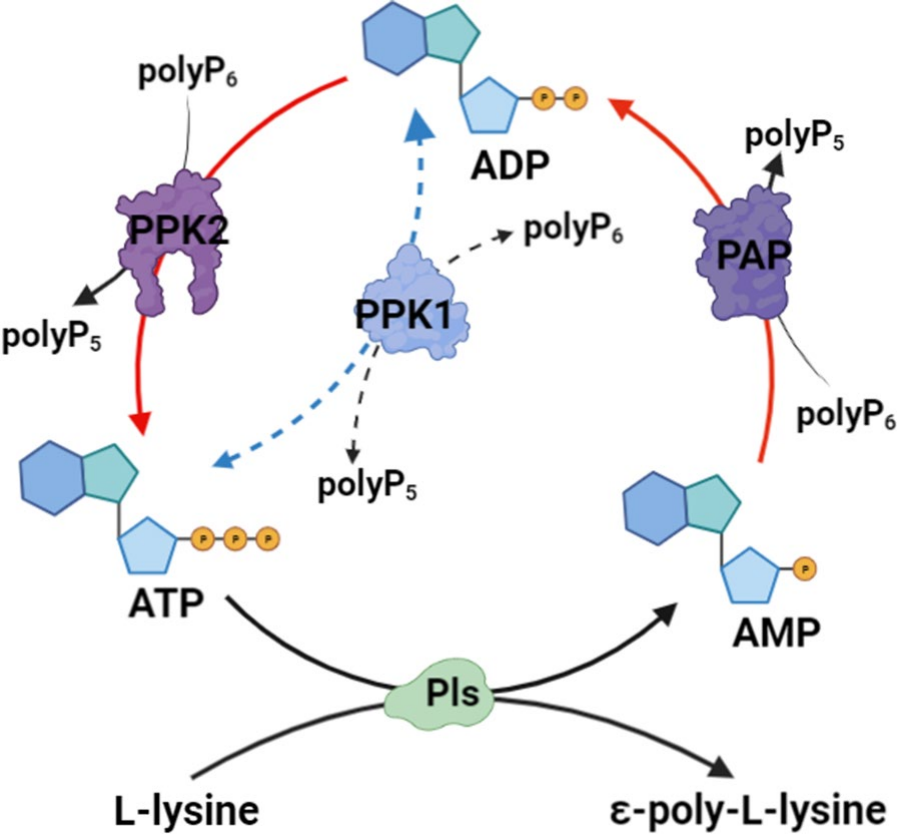
PPK-mediated ATP regeneration system coupled with ɛ-PL biosynthesis

In this study, we constructed a PPK-mediated ATP regeneration system for phosphorylating AMP to generate ATP coupled with ɛ-PL biosynthesis in the high ɛ-PL-producing strain *S. albulus* WG608 (Fig. 1). First, the effect of exogenous ATP on cell growth and ε-PL production of *S. albulus* WG608 was investigated. Then, a PPK-mediated ATP regeneration system with cascade reactions was developed, including PAP selection, PPK screening, optimal phosphate donor identification, and dynamic ATP regulation. Finally, the polyP_6_ addition parameters were optimized in batch and fed-batch fermentation. The final ε-PL production of the engineered strain PL05 was 59.25 g/L in a 5-L fed-batch fermentation, which is an increase of 33.66% compared to the ε-PL production of *S. albulus* WG608. To the best of our knowledge, this is the highest value for ε-PL production ever reported in genetically engineered *S. albulus* strains. This study not only provides guidance for robust ε-PL-producing strain modification but also serves as a reference for improving the biosynthesis of high ATP-consumption metabolites in microbes.

## Materials and methods

### Strains, plasmids, and media

All strains and plasmids used in this study are listed in Table 1. *S. albulus* WG608 is maintained by the China Center for Type Culture Collection (CCTCC M2019589) and used as a wild-type strain for ε-PL production in this study. *E. coli* DH5α and *E. coli* BL21 (DE3) were used as cloning and expression hosts, respectively. *E. coli* ET12567/pUZ8002 was used for *Streptomyces* − *E. coli* interspecies conjugation to introduce plasmids into *Streptomyces*.

**Table 1:** Strains and plasmids used in this study

Strain or plasmid	Description	Source or references
Strain		
*S.albulus*		
WG608	Parent strain, ε-poly-L-lysine producer	[29]
PL01	WG608 carrying pIB139-*pap*	This study
PL02	WG608 carrying pIB139-*ppk1*	This study
PL03	WG608 carrying pIB139-*ppk2B*^*cg*^	This study
PL04	WG608 carrying pIB139-*ppk2C*^*pa*^	This study
PL05	WG608 carrying pIB139-*ppk2B*^*cg*^*-pap*	This study
PL06	WG608 carrying pIB139-*ppk2B*^*cg*^*-pap-ppk1*	This study
*E. coli*		
DH5α	General cloning host	Invitrogen
ET12567	Donor strain for conjugation between *E.coli* and *Streptomyces*	Invitrogen
BL21(DE3)	Host for expression of PPKs	Invitrogen
BL21-pET28a-*ppk1*	BL21 carrying pET28a-*ppk1*	This study
BL21-pET28a-*ppk2B*^*cg*^	BL21 carrying pET28a-*ppk2B*^*cg*^	This study
BL21-pET28a-*ppk2C*^*pa*^	BL21 carrying pET28a-*ppk2C*^*pa*^	This study
Plasmids		
pIB39	Integrative vector based on *ϕ*C31 integrase	[30]
pIB139-*ppk1*	*ppk1* cloned in pIB139	This study
pIB139-*ppk2B*^*cg*^	*ppk2B*^*cg*^ cloned in pIB139	This study
pIB139-*ppk2C*^*pa*^	*ppk2C*^*pa*^ cloned in pIB139	This study
pIB139-*pap*	*pap* cloned in pIB139	This study
pIB139-*ppk2B*^*cg*^-*pap*	*ppk2B*^*cg*^* and pap* cloned in pIB139	This study
pIB139-*ppk2B*^*cg*^-*pap-ppk1*	*ppk2B*^*cg*^*, pap* and *ppk1* cloned in pIB139	This study
pET28a( +)	*E. coli* expression vector	Novagen
pET28a-*ppk1*	*ppk1* cloned in pET28a( +)	This study
pET28a-*ppk2B*^*cg*^	*ppk2B*^*cg*^ cloned in pET28a( +)	This study
pET28a-*ppk2C*^*pa*^	*ppk2C*^*pa*^ cloned in pET28a( +)	This study

*E. coli* strains were all cultured at 37 °C in Luria–Bertani medium (10 g/L tryptone, 5 g/L yeast powder, 10 g/L NaCl, pH 7.0) with the addition of final concentrations of 25 μg/mL kanamycin, 50 μg/mL apramycin, and 25 μg/mL chloramphenicol, if necessary. TB medium (5 g/L glycerol, 9.4 g/L K_2_HPO_4_, 2.2 g/L KH_2_PO_4_, 11.8 g/L peptone, and 23.6 g/L yeast extract) was used for protein expression. The spores of *S. albulus* WG608 and its derivatives were produced on BTN medium containing 10 g/L glucose, 2 g/L fish meal peptone, 1 g/L yeast powder, 20 g/L agar powder, pH 7.5 at 30 °C [20]. MS medium containing 20 g/L mannitol, 20 g/L soybean powder, 10 mM MgCl_2_, 20 g/L agar powder (pH 7.0) was used for intergeneric conjugation between *S. albulus* and *E. coli* at 30 °C. Moreover, the final concentrations of 50 μg/mL apramycin and 25 μg/mL nalidixic acid were overlapped on MS medium for recombinant colonies screening. YP medium containing 60 g/L glucose, 10 g/L yeast powder, 5 g/L (NH_4_)_2_SO_4_, 0.2 g/L K_2_HPO_4_·3H_2_O, 0.5 g/L MgSO_4_·7H_2_O, 0.04 g/L ZnSO_4_·7H_2_O, 0.03 g/L FeSO_4_·7H_2_O (pH6.8) was used for shake-flask fermentation of *S. albulus* derivatives [20]. YHP medium containing 60 g/L glucose, 10 g/L yeast powder, 5 g/L (NH_4_)_2_SO_4_, 4 g/L KH_2_PO_4_, 0.5 g/L MgSO_4_·7H_2_O, 0.04 g/L ZnSO_4_·7H_2_O, 0.03 g/L FeSO_4_·7H_2_O (pH6.8) was used for 5-L fermenter culture of *S. albulus* PL05 [20].

### Plasmid and strain construction

To achieve the overexpression of *pap*, *ppk1*, *ppk2B*^*cg*^ and *ppk2C*^*pa*^ in *S. albulus* WG608, the method was performed as described by our previous study [31] with some modifications*.* In specifically, the DNA fragment containing the *ppk1* coding region was amplified from the genome of *S. albulus* WG608 with the primer pair *ppk1*-F/-R, while the DNA fragments encoding *pap* (GenBank: CP068195.1), *ppk2B*^*cg*^ (Gene ID: 1020661) or *ppk2C*^*pa*^ (Gene ID: 878853) were chemically synthesized (Aenta, Suzhou, China) with codon optimization. Under the catalysis of CloneExpress II One Step Cloning Kit (Vazyme, Nanjing, China), the obtained DNA fragments were ligated into the vector pIB139 [30], respectively, which were all digested by *Nde*I and *Eco*RI. Subsequently, ligation products were then transformed into competent *E. coli* DH5α, and exconjugants were picked out from LB plates containing 50 μg/mL apramycin. After validation by colony PCR using corresponding primers and DNA sequencing (Aenta, Suzhou, China), the overexpression vectors pIB139-*pap*, pIB139-*ppk1*, pIB139-*ppk2B*^*cg*^ and pIB139-*ppk2C*^*pa*^ were obtained.

To coexpressed *ppk2B*^*cg*^ and *pap* in *S. albulus* WG608, the method was performed as described by Lv et al. [27] with some modifications. The DNA fragment containing *ppk2B*^*cg*^ was amplified from pIB139-*ppk2B*^*cg*^ by the primer pair c-*ppk2*-F/R, and the DNA fragment containing *pap* was amplified from pIB139-*pap* by the primer pair c-*pap*-F-2/-R-2. The two fragments were ligated into the vector pIB139 that was digested with *Nde*I and *Eco*RI to obtain the co-expression vector pIB139-*ppk2B*^cg^-*pap*.

For the coexpression of *ppk2B*^*cg*^, *pap* and *ppk1* in *S. albulus* WG608*,* the DNA fragment containing *ppk2B*^*cg*^ was amplified from pIB139-*ppk2B*^*cg*^ by the primer pair c-*ppk2*-F/R, the DNA fragment containing *pap* was amplified from pIB139-*pap* by the primer pair c-*pap*-F-3/-R-3, and the DNA fragment containing *ppk1* was amplified from pIB139-*ppk1* by the primer pair c-*ppk1*-F/-R. The three fragments were ligated into the vector pIB139 that was digested with *Nde*I and *Eco*RI to obtain the co-expression vector pIB139-*ppk2B*^cg^-*pap-ppk1*. All primers are listed in Additional file 1: Table S1.

Finally, the above recombinant plasmids were separately transformed into *E. coli* ET12567 for intergeneric conjugation with *S. albulus* WG608. To obtain overexpression strains, the transformants were screened on BTN solid medium supplemented with apramycin and nalidixic acid, and the colonies were verified by PCR using the corresponding primers.

### Protein expression, purification, and quantification

To facilitate the expression of PPKs, *ppk1*, *ppk2B*^*cg*^, and *ppk2C*^*pa*^ were ligated to pET28a( +) containing a 6 × His tag, respectively. Plasmids pET28a-*ppk1*, pET28a-*ppk2B*^*cg*^*,* and pET28a-*ppk2C*^*pa*^ were obtained. The above recombinant plasmids were transferred into competent *E. coli* BL21(DE3) to obtain recombinant strains.

For protein expression, the recombinant *E. coli* strains were incubated overnight at 37 °C in LB medium and then transferred to TB medium. When OD_600_ reached 0.6–0.8, 0.5 mM isopropyl-β-d-thiogalactopyranoside (IPTG) was added and incubated at 30 °C for 8–12 h. Then, cells were collected by centrifugation (4 ℃, 10000 rpm, 10 min) using the high-speed refrigerated centrifuge and washed twice with 50 mM Tris–HCl buffer (pH 7.5). Cells were 10 times concentrated with 50 mM Tris–HCl buffer (pH 7.5) and followed by sonication disruption at the output level of 60%. The operating time was set as 1 s with a 3 s rest interval, and the crushing time is 30 min, and cell debris was removed by centrifugation at 12000 rpm for 10 min and the supernatant was collected as crude enzyme solution. The proteins were purified from the crude enzyme solution by nickel affinity chromatography on AKTA avant 25 (GE Healthcare, USA) using Wash Buffer A (20 mM Tris–HCl, pH 7.5, 30 mM imidazole and 500 mM NaCl) and Elution Buffer B (20 mM Tris–HCl, pH 7.5, 500 mM imidazole and 500 mM NaCl). Protein concentrations were determined using TaKaRa Bradford Protein Assay Kit (TaKaRa, Japan) using bovine serum albumin as a standard. Sodium dodecyl sulfate (SDS)-polyacrylamide gel electrophoresis (PAGE) analysis was performed using a 10% bis–tris protein gel stained with Komas Brilliant Blue solution.

### Determination of PPK activity

In order to compare the ATP synthesis capacity of different sources of PPKs, the reaction system (1 mL) consisted of 50 mM Tris–HCl (pH 8.0), 20 mM MgSO_4_, 5 μM ADP, 5 μM polyP_n_ (different polymerization degrees) and 100 μg of PPKs was adopted [32]. The mixture was equilibrated at 30 °C for 5 min and 100 μg of enzyme solution was given to initiate the reaction. After incubation at 30 °C for 2 h, the reaction was terminated by the addition of 0.1% (v/v) trifluoroacetic acid. The ATP concentration in the reaction system was measured and the regeneration capacity of ATP was expressed as the amount of ATP formed by 1 mg of enzyme catalysis in 1 h at 30 °C.

To investigate the reversible reaction of PPK1, a 1 mL reaction system containing 50 mM Tris–HCl (pH 8.0), 20 mM MgSO_4_, 5 μM ADP, 5 μM polyP_6_, 100 μg of purified PPK1 kinase, with different concentrations of ATP (0–10 μM) was used. The mixture was also equilibrated at 30 °C for 5 min and 100 μg of enzyme solution was given to initiate the reaction. After incubation at 30 °C for 2 h, the reaction was terminated by the addition of 0.1% (v/v) trifluoroacetic acid. The ATP concentration was measured and the change of ATP concentration was expressed as the amount of ATP formed or consumed by 1 mg of PPK1 catalysis in 1 h at 30 °C.

### Measurement of intracellular ATP and ADP

For the measurement of intracellular ATP, the method was performed as described by Yan et al. [33] with some modifications. The intracellular ATP was measured using a luciferin/luciferase method using an ATP Assay Kit (Beyotime, Jiangsu, China) according to the manufacturer's instructions. In brief, cells were treated with the ATP assay lysate in the kit at 4 °C for 15 min to break the cells and centrifuged at 12,000 g for 5 min at 4 °C to prepare supernatants for ATP and ADP testing. An ATP concentration standard curve was generated using a series of known concentrations of ATP standard solutions (1 nM*–*1 μM). Subsequently, 100 μL ATP assay buffer was added to each well of black 96-well plate, incubated for 5 min before the addition of 100 μL sample to each well, mixed, and luminescence was measured by a Hybrid Multi-Mode reader (Bio-Tek Instruments, Inc, Winooski, VT, USA). Then, the concentration of ATP was calculated according to the standard curve. ATP content is determined by unit protein content.

The intracellular ADP was measured using a double antibody one-step sandwich enzyme-linked immunosorbent assay (ELISA) using a microorganism adenosine diphosphate (ADP) ELISA Kit (Beyotime, Jiangsu, China) according to the manufacturer's instructions. Adenosine diphosphate (ADP) antibodies were added to the precoated microtiter wells, and the sample, standard and HRP-labeled detection antibody were added sequentially, warmed and washed thoroughly. TMB is converted to blue by peroxidase and to the final yellow by the action of acid. The shade of color was positively correlated with the amount of adenosine diphosphate (ADP) in the sample. The absorbance (OD value) was measured by a Hybrid Multi-Mode reader (Bio-Tek Instruments, Inc, Winooski, VT, USA) at 450 nm. An ADP concentration standard curve was generated using a series of known concentrations of ADP standard solutions (0*–*4800 nM). Then, the concentration of ADP was calculated according to the standard curve. ADP content is determined by unit protein content.

### Shake-flask, batch and fed-batch fermentation of *S. albulus*

In the shake-flask fermentation, *S. albulus* WG608 and its derivatives were inoculated in YP medium and incubated at 30 °C, 200 rpm for 24 h as seed culture, then the seeds were inoculated into YP medium at an 8% (v/v) inoculum and continued to be incubated at 30 °C, 200 rpm for 72 h.

In the batch fermentation the method was performed as described by Pan et al*.* [34] with some modifications. The batch fermentations were performed in a 1-L glass bioreactor system (T&J Bio-Engineering Co. Ltd., Shanghai, China) consisting of four identical stirred tank reactors with a filling volume of 0.7 L. The broth was agitated at 300 rpm with a mechanical stirrer at 30 °C with a 1 vvm aeration rate. The initial pH was adjusted to 6.80 by adding ammonia water (12.5%, v/v), and then the broth was inoculated with 60 mL of 24-h-old seed culture. During the fermentation, pH and DO were also monitored online using pH and DO electrodes, respectively (K8S-120 and InPro6800-12–120, Mettler Toledo, Zurich, Switzerland). When the pH spontaneously dropped to 4.0 from the initial pH of 6.80, pH of 4.0 was maintained by automatically adding ammonia water (12.5%, v/v) until the end of fermentation. The DO was maintained above 30% of air saturation before pH declined to 4.0; afterward, the DO was maintained above 20% of air saturation until the end of fermentation, which was controlled by adjusting agitation speed from 200 to 1000 rpm. When agitation speed reached 800 rpm, aeration rate was then manually increased stepwise with steps of 0.5 vvm and a range of 1–3 vvm. In addition, 1 g/L of polyP_6_ was added to the medium before fermentation. Fermentation ends when the carbon source in the medium is depleted.

In the fed-batch fermentation, the method was performed as described by Pan et al*.* [34] with some modifications. Fermentation in a 5-L glass stirred tank bioreactor (Biotech-5BG, Baoxing Bio-Engineering Equipment Co., Ltd., Shanghai, China) was performed by filling in 3.5 L YHP medium. Before the inoculation, the sterilized bioreactor’s temperature, aeration rate, and agitation speed were maintained at 30 °C, 0.5 vvm, and 200 rpm, respectively, and the initial pH was controlled at 6.8 by the manual addition of ammonia water (12.5%, w/v). When the system was stable, 320 mL of 24-h-old seed culture was inoculated into the bioreactor. During the fermentation, pH and DO were monitored online using pH and DO electrodes (K8S-225 and InPro6800-12–220, Mettler Toledo, Zurich, Switzerland), respectively. The control of DO and pH was the same as that in the above mentioned 1-L batch fermentation. However, the agitation speed was varied from 200 to 900 rpm, and aeration rate was manually increased stepwise with steps of 0.5 vvm and a range of 0.5–2.5 vvm. In addition, 1 g/L of polyP_6_ was added to the medium before fermentation. The intracellular ATP content was measured every 12 h, when the intracellular ATP content decreased significantly, 1 g/L of polyP_6_ was added manually for preventing phosphate limitation. Moreover, when the glucose concentration in the fermentation was below 10 g/L, sterilized glucose (80%, w/v) was automatically added and maintained at about 10 g/L for preventing carbon source limitation. When the ammonia nitrogen (NH_4_^+^-N) concentration decreased below 0.5 g/L, sterilized (NH_4_)_2_SO_4_ solution (40%, w/v) was automatically added and maintained at about 0.5 g/L for preventing nitrogen source limitation.

### Fermentation parameters analysis

Determination of fermentation parameters was performed as described by Ren et al. [35] with some modifications. DCW determination: 10 mL aliquots of culture broth was subjected to centrifugation at 12000 rpm for 10 min, and then the precipitate was collected. The mycelia were filtered through a pre-weighed filter paper and dried at 105 °C to a constant weight prior to measuring the biomass. The centrifugal supernatant was used to determine the ε-PL concentration according to the procedure described by Itzhaki [36]. Glucose concentration was determined by a biosensor (SBA-40B, Shandong Academy of Sciences, China) through the enzymatic reaction of glucose oxidase. NH_4_^+^-N was analyzed by means of a colorimetric method using Nessler reagent [37].

### Statistical analysis

All experiments were conducted 3 times, and all data were expressed as mean ± standard deviation. The SPSS (version 22.0, SPSS Inc., Chicago, ILL, USA) was used for statistical analysis that was performed using one-way analysis of variance (ANOVA) and Tukey’s test at p < 0.05.

## Results

### Effect of exogenous ATP on cell growth and ε-PL production of *S. albulus* WG608

**Fig. 2:**
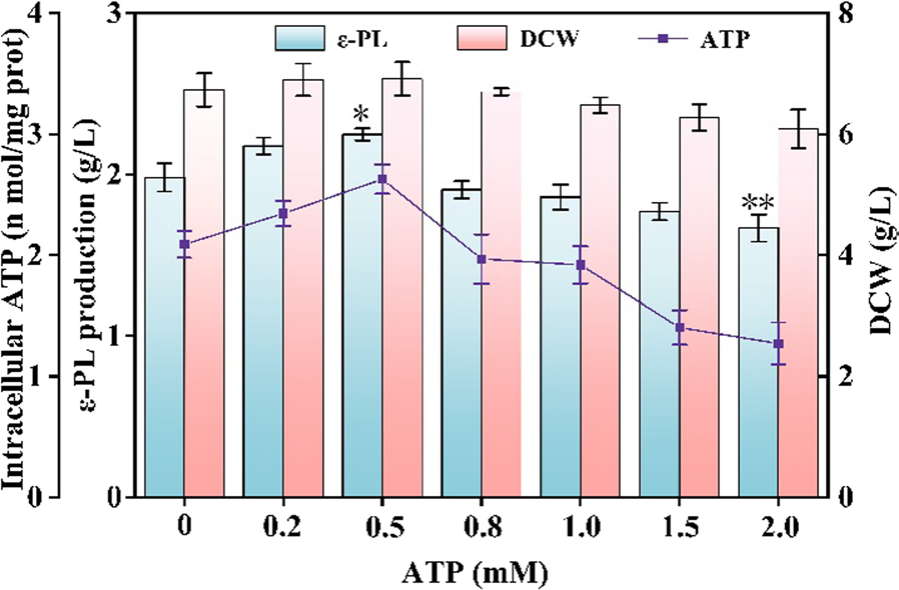
Effect of exogenous ATP on cell growth and ε-PL production of *S. albulus* WG608. The data are presented as averages, and the error bars represent standard deviations (n = 3 independent experiments). ^*^0.01 < P < 0.05, ^**^P < 0.01

To investigate changes in cell growth and ε-PL production of *S. albulus* WG608, ATP was directly added to the culture medium at final concentrations ranging from 0.2 mM to 2.0 mM. As shown in Fig. 2, ε-PL production and the intracellular ATP concentration were increased in the presence of 0.2 mM to 0.5 mM ATP and decreased when exogenous ATP was in excess of 0.8 mM. The highest ε-PL production was 2.25 g/L at 0.5 mM ATP, which was 13.64% higher than the ε-PL production of the control. Moreover, the intracellular ATP concentration was increased by 25.86%. However, ε-PL production and the intracellular ATP concentration were reduced by 15.66% and 38.94%, respectively, when the final exogenous ATP concentration was 2.0 mM. Interestingly, the cell growth of *S. albulus* WG608 was not affected by exogenous ATP at the tested concentrations and only decreased slightly with exogenous ATP in the range of 1.0 mM to 2.0 mM. These results show that ε-PL production was enhanced, more or less, by the addition of a relatively low concentration of ATP, whereas the addition of a relatively high concentration of ATP led to a decrease in ε-PL production of *S. albulus* WG608.

### Construction of the ATP regeneration system in *S. albulus* WG608

**Fig. 3:**
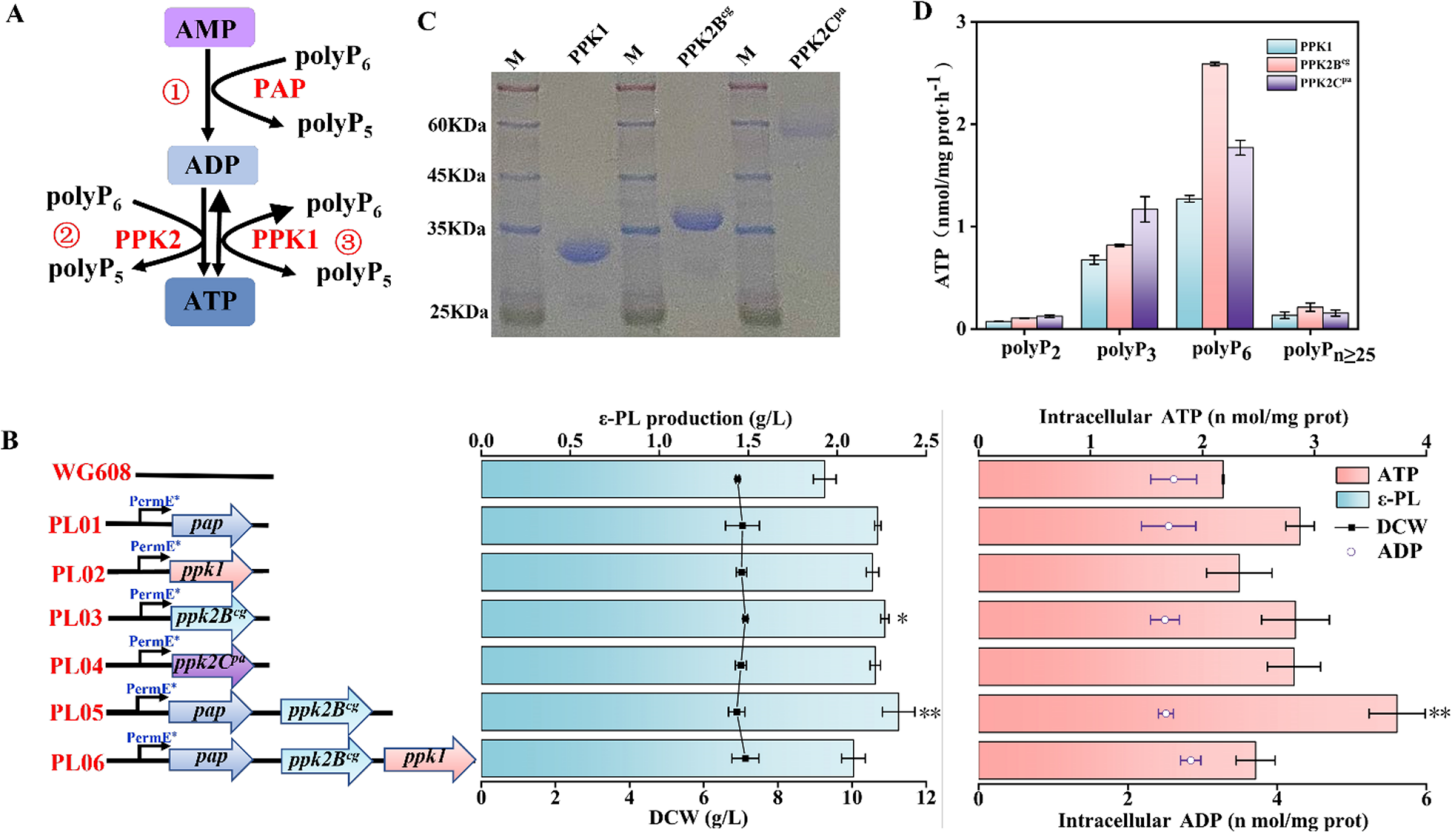
Purification of PPK and comparison of ATP regeneration capacity. **A**: Model of ATP regeneration system. **B**: Shake‒flask fermentation of *ppk* heterologously expression strains. **C**: SDS‒PAGE analysis of the purification of PPKs. **D** Specific enzyme activity assay of different PPKs using different polyP_n_. The data are presented as averages, and the error bars represent standard deviations (n = 3 independent experiments). ^*^ 0.01 < P < 0.05, ^**^ P < 0.01

During ε-PL biosynthesis, ATP is used for adenylation of the precursor L-lysine and converted into AMP. Thus, we proposed a cascade reaction for ATP regeneration from AMP in *S. albulus* WG608 that included two steps: (i) rephosphorylating the AMP to generate ADP catalyzed by PAP and (ii) rephosphorylating the ADP to generate ATP catalyzed by PPK using polyP as an external phosphate donor (Fig. 3A). PAPs from *Acinetobacter johnsonii* and *Myxococcus xanthus* can rephosphorylate AMP to generate ADP, but PAP from *A. johnsonii* exhibits its highest activity in the presence of Mg^2+^, and PAP from *M. xanthus* exerts its highest activity in the presence of Mn^2+^ [38]. Considering the presence of Mg^2+^ in our medium, we chose PAP from *A. johnsonii* to catalyze the conversion of AMP into ADP. As a result, the gene *pap* encoding PAP from *A. johnsonii* was introduced into the chromosome of *S. albulus* WG608 and strain PL01 was generated. As shown in Fig. 3B, the ε-PL production of strain PL01 reached 2.23 ± 0.02 g/L in shake-flask fermentation, which was 15.54% higher than the ε-PL production of *S. albulus* WG608. Additionally, the intracellular ATP was increased by 31.65% compared with that of *S. albulus* WG608.

PPKs have been classified into two families, PPK1 and PPK2, which catalyze the conversion of ADP into ATP. As described in a previous study, PPKs from different microbes displayed varying activities on different polyP substrates [32]. Herein, PPK1 from *S. albulus* WG608 itself and PPK2 from *Corynebacterium glutamicum* (PPK2B^cg^) and *Pseudomonas aeruginosa* (PPK2C^pa^) were expressed in *E. coli* BL21(DE3) and purified by nickel-affinity chromatography (Fig. 3C). The activities of the three PPKs were compared in vitro using ADP, and different polyPs, which were polymerized to different degrees, as substrates. As shown in Fig. 3D, the three PPKs showed varying ATP synthesis activities on different polyP substrates, and they had the highest specific activity in response to polyP_6_. In particular, PPK2B^cg^ exhibited the highest ATP production ability. To investigate the ATP regeneration ability of the three PPKs from ADP in *S. albulus* WG608, the genes *ppk1*, *ppk2B*^*cg*^ and *ppk2C*^*pa*^ were introduced into the chromosome of *S. albulus* WG608 to obtain strains PL02, PL03 and PL04, respectively (Fig. 3B). The ε-PL yields of strains PL02, PL03 and PL04 reached 2.20 ± 0.04 g/L, 2.27 ± 0.02 g/L, and 2.21 ± 0.04 g/L, respectively, in shake-flask fermentation, which were 13.99%, 17.61% and 14.51% higher than the ε-PL yields of *S. albulus* WG608, respectively. Moreover, the intracellular ATP contents in the three recombinant strains were increased by 6.88%, 29.82% and 28.90% compared with the intracellular ATP contents of *S. albulus* WG608, respectively. These results demonstrate that *ppk2B*^*cg*^ is the superior PPK used for ATP regeneration from ADP in *S. albulus* WG608.

To construct the whole ATP regeneration system, we coexpressed *pap* and *ppk2B*^*cg*^ in *S. albulus* WG608 to obtain strain PL05, which has the whole ATP regeneration system with the reaction cascade of AMP to ADP and ADP to ATP (Fig. 3B). Strain PL05 produced 2.34 ± 0.09 g/L ε-PL in shake-flask fermentation, which is an increase of 21.24% compared with ε-PL production of *S. albulus* WG608. Moreover, the intracellular ATP was also increased by 71.56%.

### Construction of an ATP regeneration system with ATP degradation in *S. albulus* WG608

**Fig. 4:**
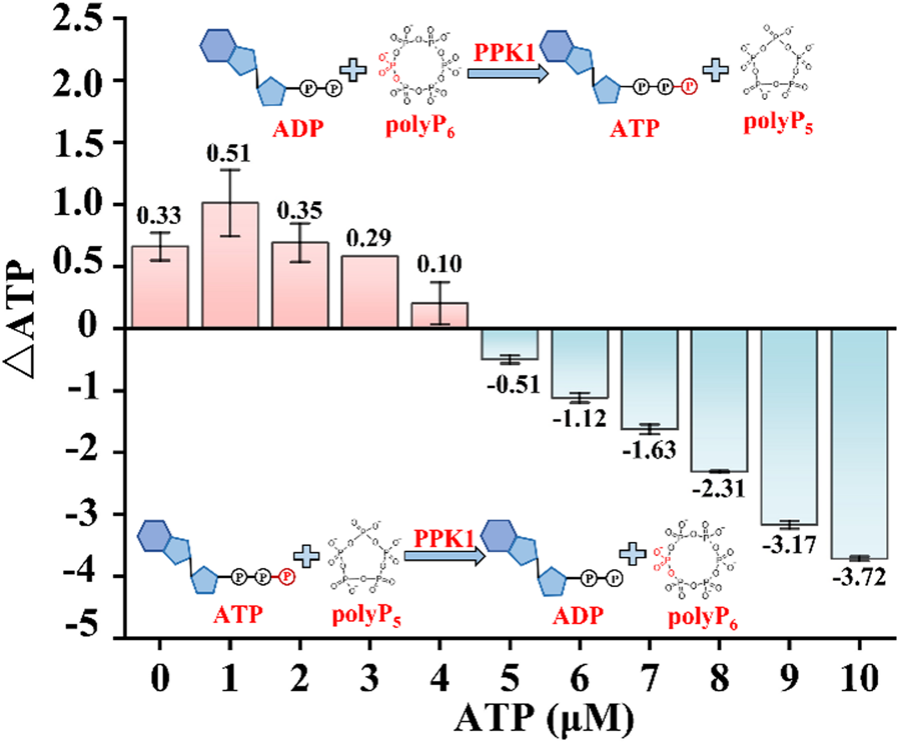
PPK1 reversibly catalyzes the dynamic balance of ADP and ATP. The data are presented as averages, and the error bars represent standard deviations (n = 3 independent experiments)

It has been reported that PPK1 catalyzes the reversible reaction converting ADP into ATP. However, what the balance depends on is not clear. Therefore, the ATP concentrations were determined in an in vitro Tris–HCl (pH 8.0) buffer system containing 100 μg of purified PPK1, 5 μM polyP_6_ and 5 μM ADP with 0 ~ 10 μM ATP. As shown in Fig. 4, when the added ATP concentration is lower than the ADP concentration (5 μM), PPK1 catalyzes polyP_6_ depolymerization and ATP is produced; however, when the added ATP concentration is higher than the ADP concentration (5 μM), PPK1 catalyzes polyP_6_ polymerization and ATP is degraded into ADP. To avoid excessive intracellular ATP damaging ε-PL biosynthesis or cell growth, an ATP degradation route was introduced into the constructed ATP regeneration system (Fig. 3A). As a result, the *ppk1* gene (encoding PPK1) combined with *pap* and *ppk2B*^*cg*^ was coexpressed in *S. albulus* WG608 to generate strain PL06 (Fig. 3B). The ε-PL production of strain PL06 was only increased by 8.29% compared with that of *S. albulus* WG608. Moreover, the intracellular ATP content of strain PL06 was reduced by 33.96% compared with that of strain PL05.

### Effect of the polyP_6_ concentration on ε-PL production by strain PL05

**Fig. 5:**
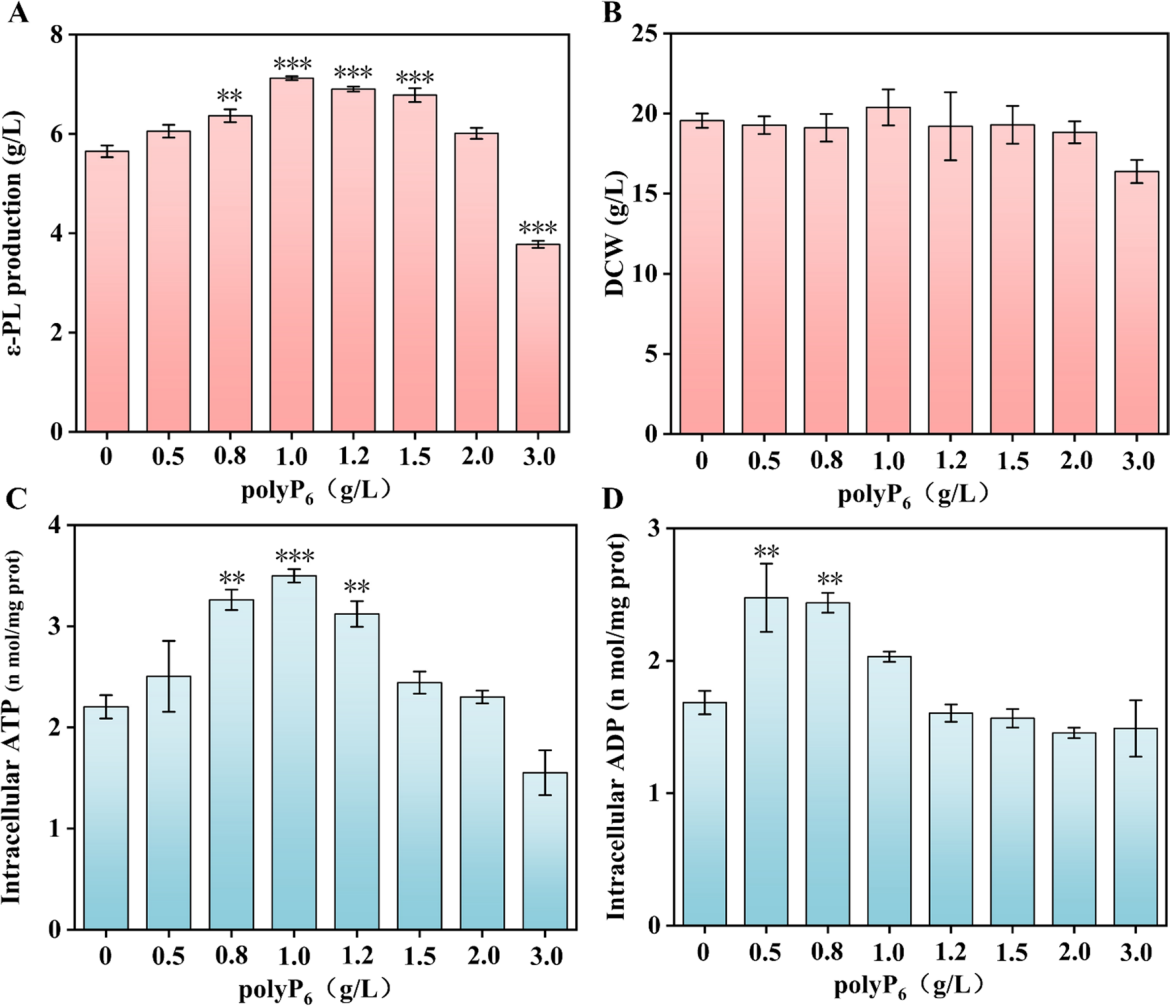
Effect of polyP_6_ concentration on ε-PL production by strain PL05 in batch‒fermentation. **A**: ε-PL production. **B**: Biomass. **C**: Intracellular ATP content. **D**: Intracellular ADP content. The data are presented as averages, and the error bars represent standard deviations (n = 3 independent experiments). ^*^ 0.01 < P < 0.05, ^**^ P < 0.01, ^***^ P < 0.001

PolyP_6_ is the optimal phosphate donor for the ATP regeneration system; thus, it is imperative to study the effect of the polyP_6_ concentration on ε-PL production by strain PL05. As shown in Fig. 5, the concentration of the substrate polyP_6_ significantly affected ε-PL production and intracellular ATP/ADP of strain PL05 in batch fermentation, but had a slight influence on cell growth (Fig. 5B). The ε-PL production of strain PL05 increased in the presence of 0.5 g/L to 1.0 g/L polyP_6_ and decreased when polyP_6_ was in excess of 1.2 g/L (Fig. 5A). The highest ε-PL production of 7.15 g/L was attained with the addition of 1.0 g/L polyP_6_, which was an increase of 28.37% compared with the control. The intracellular ATP of strain PL05 increased considerably with 0.5 g/L–1.0 g/L polyP_6_ and significantly decreased when polyP_6_ was in excess of 1.2 g/L (Fig. 5C); these results were highly consistent with changes in ε-PL production. In addition, intracellular ADP was increased by the addition of 0.5 g/L–0.8 g/L polyP_6_ and decreased when polyP_6_ was in excess of 1.0 g/L (Fig. 5D). Taken together, the optimal concentration of substrate polyP_6_ for ε-PL production by strain PL05 in batch fermentation was 1.0 g/L.

### Production of ε-PL in fed-batch fermentation by strain PL05 with polyP_6_ addition

**Fig. 6:**
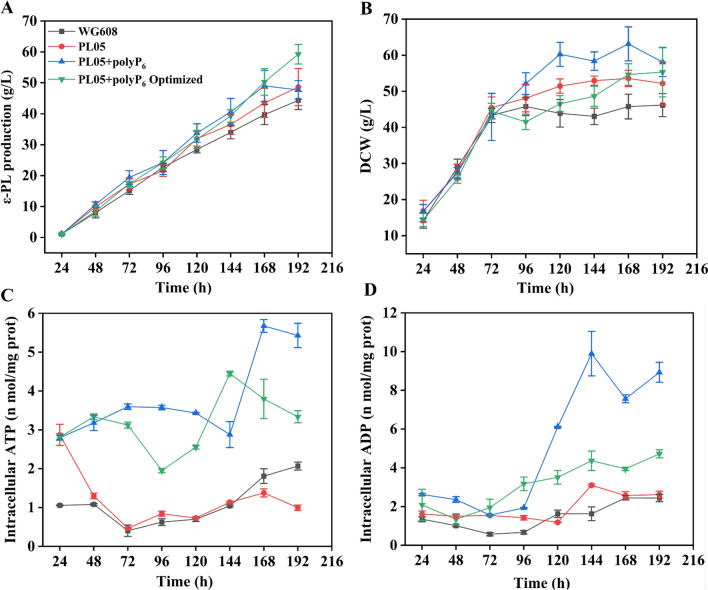
Optimization of polyP_6_ addition strategy in the fed-batch fermentation of strain PL05. **A**: ε-PL production. **B**: Biomass. **C**: Intracellular ATP content. **D**: Intracellular ADP content. PL05 + polyP_6_: strain PL05 with polyP_6_ addition at the final concentration of 1 g/L every 48 h. PL05 + polyP_6_ Optimized: strain PL05 with polyP_6_ addition at the final concentration of 1 g/L at 0 h and 108 h. The data are presented as averages, and the error bars represent standard deviations (n = 3 independent experiments)

To achieve the maximum ε-PL production by strain PL05, a fed-batch fermentation in a 5-L fermenter was adopted (Additional file 1: Fig. S1). As shown in Fig. 6A, the ε-PL production of strain PL05 without polyP_6_ addition was 48.62 g/L at 192 h, which was an increase of 9.68% compared to the ε-PL production of strain WG608. When polyP_6_ was added to the culture medium at a concentration of 1 g/L every 48 h, the ε-PL production of strain PL05 was increased after 48 h and the maximum ε-PL production of 49.01 g/L was attained after 168 h. However, ε-PL production ceased to increase in the last 24 h of fermentation and even decreased. The analyses of intracellular ATP and ADP levels showed that the ATP content increased after 144 h and peaked at 168 h, while the ADP content increased sharply from 96 h and peaked at 144 h (Fig. 6C, D). To avoid the dramatic increase in intracellular ATP and ADP, we optimized the strategy of polyP_6_ addition during fermentation based on intracellular ATP measurements every 12 h: polyP_6_ was added at a final concentration of 1 g/L at 0 h and 108 h. The increase in ε-PL production continued through the final 24 h of fermentation and the highest ε-PL production of 59.25 g/L was attained at 192 h. Interestingly, the DCW of strain PL05 was significantly increased with or without polyP_6_ supplementation compared with that of strain WG608 (Fig. 6B). In particular, the highest DCW was attained when polyP_6_ was added to the culture medium every 48 h. However, the DCW was reduced significantly after adding the optimized amount of polyP_6_, which is beneficial for downstream processes. As shown in Fig. 6C, D, the intracellular ATP and ADP levels of strain PL05 without polyP_6_ addition were comparable with those of strain WG608, whereas ATP and ADP levels were dramatically enhanced when polyP_6_ was added. Moreover, the changes in intracellular ATP and ADP levels in the optimized process were more stable than those in the processes before optimization.

## Discussion

ATP exists in all living organisms and acts as the molecular currency of intracellular energy. It provides energy for chemical reactions and participates directly in the phosphorylation of regulatory proteins [39]. Moreover, it also plays a regulatory role in animal tissues [40] and plant physiology [41]. During ε-PL biosynthesis, ATP is used as an activator for the adenylation of precursor L-lysine catalyzed by ε-PL synthetase (Pls), which is a nonribosomal peptide synthase located in the cell membrane [42]. Moreover, Yamanaka et al. [18] found that the catalytic function of Pls is strictly regulated by intracellular ATP, and a high concentration of ATP is required for full enzymatic activity. As a result, the intracellular ATP concentration is a key regulator and energy substrate of ε-PL biosynthesis.

In this study, we first investigated the effects of exogenous ATP on ε-PL production and cell growth of *S. albulus* during fermentation. The results showed that a low concentration of exogenous ATP (0.2 ~ 0.5 mM) could significantly improve the ε-PL production of *S. albulus* WG608, but a high concentration of exogenous ATP (1.0 ~ 2.0 mM) decreased ε-PL production and even cell growth (Fig. 1). Interestingly, the relationship between intracellular ATP and exogenous ATP concentrations was highly consistent with that between ε-PL production and exogenous ATP. These results are consistent with the results reported by Meng et al. [43], in which, based on the evaluation of four *Streptomyces* strains, low extracellular ATP levels stimulated antibiotic production and high extracellular ATP levels reduced antibiotic production. Due to its size and charge, ATP cannot cross the cell membrane and enter the cytoplasm without a transport protein [44]. If there is a transport system, adding exogenous ATP should result in a dose-dependent increase in intracellular ATP. However, in this study, we observed that a high concentration of exogenous ATP addition decreased intracellular ATP significantly, indicating that a high concentration of exogenous ATP serves as a stimulus that alters the innate homeostatic balance of intracellular ATP. Therefore, Li et al. [45] suggested that exogenous ATP, similar to phosphorylated guanosine nucleotide, *S*-adenosyl-L-methionine, and cyclic AMP, works as a regulatory molecule in *Streptomyces*. Considering the cost of ATP used on an industrial scale and its contribution to ε-PL production, exogenous ATP addition might not be an economical or effective strategy for enhancing ε-PL production.

Another method for increasing intracellular ATP concentration is to construct an ATP regeneration system for generating ATP from AMP. To date, researchers have established several systems for synthesizing ATP from ADP, including the use of acetate kinase-catalyzed acetyl phosphate, pyruvate kinase-catalyzed phosphoenolpyruvate, creatine kinase-catalyzed phosphocreatine, and carbamate kinase-catalyzed carbamoyl phosphate [25]. Moreover, several systems for synthesizing ATP from AMP have also been developed. However, most AMP-to-ATP regeneration systems employ two enzymes (such as adenylate kinase/acetate kinase, adenylate kinase/PPK, or two-PPK systems) or more complicated mixtures, such as *E. coli* lysate supplemented with acetyl phosphate and whole cells [26]. In the present study, we proposed a cascade of reactions with two steps, AMP → ADP and ADP → ATP (Fig. 3A). First, we selected PAP (class II PPK2) from *A. johnsonii* and tested it in *S. albulus* WG608 for catalyzing AMP to ADP (strain PL01). Then, we confirmed that PPK2B^cg^ from *Corynebacterium glutamicum* is superior for catalyzing the conversion of ADP into ATP through in vitro and in vivo experiments. Finally, PAP and PPK2B^cg^ were subsequently coexpressed in *S. albulus* WG608 to construct strain PL05 with a complete system to regenerate ATP. Predictably, the recombinant strain PL05 achieved the highest ε-PL production of 2.34 g/L in shake-flask fermentation, which was an increase of 21.24% compared with the ε-PL production of *S. albulus* WG608; intracellular ATP was also increased by 71.56%. To the best of our knowledge, this is the first report on enhancing ε-PL production by constructing an ATP regeneration system. In fact, the above ε-PL production of strain PL05 is not satisfactory, which might be caused by the absence of polyP_6_ and an uncontrolled pH during shake-flask fermentation. Therefore, we systemically evaluated the effect of polyP_6_ addition on the ε-PL production of strains PL01 ~ PL05 by batch fermentation in a 1-L fermenter with a constant pH of 4.0 (Additional file 1: Fig. S2). Unfortunately, the ε-PL production of the recombinant strains, including strain PL05, was not significantly changed compared with that in shake-flask fermentation.

The other possible reason for the unsatisfactory ε-PL production of strain PL05 is that excessive intracellular ATP impairs secondary metabolite biosynthesis [19]. To address this issue, it is necessary to introduce the ATP degradation pathway into strain PL05. It has been reported that PPK1 catalyzes the polymerization of polyP from γ-phosphate derived from ATP, while PPK1 from *E. coli* catalyzes ATP synthesis, indicating that PPK1 can reversibly catalyze the phosphorylation reaction between ADP and ATP [46]. Kamatani et al. [47] reported that, in the presence of 0.2 mM polyP_60-70_, if the proportion of ADP in the mixture of ADP and ATP exceeded one-third, the equilibrium of the phosphorylation reaction between ADP and ATP catalyzed by PPK1 shifted to ATP synthesis. In this study, we confirmed that PPK1 derived from *S. albulus* WG608 also has the ability to reversibly catalyzed the reaction between ADP and ATP. When the concentration of ATP is higher than that of ADP, the reaction is directed to ADP formation, but when the concentration of ADP is higher than that of ATP, the reaction is directed to ATP formation (Fig. 3). Therefore, an ATP degradation reaction catalyzed by PPK1 was introduced into strain PL05, thus generating strain PL06. Unexpectedly, the ε-PL production of strain PL06 did not show an improvement, although the intracellular ATP level was significantly lower than that of strain PL05. This is presumably because the ATP regeneration system implemented in strain PL05 was not causing the intracellular ATP concentration to reach a level unfavorable to ε-PL production or cell growth.

To ensure that the ATP regeneration system works properly, polyP needs to be provided to the recombinant strain PL05 during fermentation under appropriate conditions. According to the in vitro PPK activity assay (Fig. 3D) and the optimization of polyP addition in the batch fermentation of strain PL05, we found that the highest intracellular ATP and ε-PL production were achieved with a final polyP_6_ concentration of 1 g/L. Because it is difficult to determine the concentration of polyP_6_ in the fermentation broth, we first added 1 g/L polyP_6_ into the culture medium every 48 h in the process of fed-batch fermentation from the beginning of fermentation. Unexpectedly, the maximum ε-PL production of 49.01 g/L was nearly equivalent to that without polyP_6_ addition. We speculated that the large amount of intracellular ATP accumulation (due to excessive polyP_6_ addition) might be responsible for the cessation of ε-PL biosynthesis in the last 24 h of fermentation. Therefore, based on intracellular ATP measurements every 12 h, we changed the time of 1 g/L polyP_6_ addition to 0 h and 108 h during fed-batch fermentation (Additional file 1: Fig. S1). As expected, the accumulation of ε-PL was recovered in the last 24 h of fermentation and the highest ε-PL production of 59.25 g/L was achieved after 192 h, which is an increase in ε-PL production of 33.66% compared with the control. Thus, we conclude that the addition of polyP_6_ to engineered *S. albulus* strains with an ATP regeneration system is vital for high-yield ε-PL production. As illustrated in Table 2, this is the highest value for ε-PL production by a genetically engineered *S. albulus* strain in fed-batch fermentation, demonstrating that this engineered *S. albulus* strain is viable for ε-PL production.

**Table 2:** Overview on the production of ε-PL by engineered *S. albulus* in fed-batch fermentation

Strain	Engineered strategy	Fermentation strategy	Production (g/L)	Productivity (g/L/day)	References
Mutagenesis and resistance screening					
*S. albulus* 11011A	AEC and Gly resistance screening	Constant pH 4.0	20	4	[48]
*S. albulus* S410	–	Two-stage pH control	48.3	6.04	[49]
* S. albulus* F3-4	Genome shuffling	Constant pH 3.8	13.5	2.23	[50]
* S. albulus* PD-1	Wild-type	Oxygen-vectors addition	30.8	4.4	[22]
* S. albulu*s FEEL-1	Genome shuffling and ribosome engineering	Constant pH 4.0	24.5	3.5	[51]
* S. albulus* F4-22	Genome shuffling	Constant pH 4.0	39.96	5.54	[52]
* S. albulus* AG3-28	Genome shuffling and gentamicin-resistance screening	Constant pH 3.8	56.5	5.65	[53]
* S. albulus* M-Z18	UV and NTG mutagenesis	pH shock	32.22	5.86	[54]
* S. albulus* R6	ARTP mutagenesis and multiple antibiotic-resistance screening	pH shock	70.3	8..01	[20]
* S. albulus* GS114	Streptomycin resistance screening	Dynamic pH regulation	60.2	7.5	[55]
Genetic engineering					
*S. albulus* CR1-*ask*	Site-directed mutation of Ask	Constant pH 4.0	15	2.14	[56]
*S. albulus* PD-2	Overexpressed *vgb*	Two-stage pH control	34.2	4.89	[23]
*S. albulus* NK660	Overexpressed *vgb* and *metK*	Two-stage pH control	0.76	0.25	[57]
*S. albulus* PD-1-*amtB*	Overexpressed *amtB*	Two-stage pH control	35.7	5.1	[58]
*S. albulus* PD-5	Knock-out *pls*I and expressed *pls*II	Two-stage pH control	23.6	3.83	[59]
*S. albulus* NBRC14147	Overexpressed *ttm* and *nys*	Two-stage pH control	3.5	2.8	[60]
*S. albulus* Q-PL2	Overexpressed *pls*	Constant pH 4.0	20.1	6.7	[4]
*S. albulus* PL05	Introduced PPK-mediated ATP regeneration system	Constant pH 4.0	59.25	7.41	This study

In recent decades, mutagenesis and resistance screening have been the dominant methods for engineering ε-PL-producing strains [13]. Although several high-yield strains have been bred for industrial ε-PL production, mutagenesis and resistance screening is time-consuming, laborious and random. With the development of omics technology and synthetic biology, the pathway to biosynthesize ε-PL has become increasingly clear, which provides a foundation for the construction of ε-PL cell factories using rational metabolic engineering and synthetic biology. The study presented here is an example. Future studies could focus on strengthening the L-lysine synthesis pathway, weakening or eliminating branch metabolism, and highly expressing *pls* to generate next-generation high-yield ε-PL-producing strains.

## Conclusions

In this study, a new *S. albulus* strain was engineered by introducing a PPK-mediated ATP regeneration system coupled with ε-PL biosynthesis to enhance ε-PL production. Low-dose exogenous ATP addition could improve ε-PL production, suggesting that an increase in ATP supply is beneficial for ε-PL biosynthesis. Through an in vivo test, PAP from *A. johnsonii* was selected for catalyzing the conversion of AMP into ADP. Moreover, three PPKs were compared by in vitro and in vivo studies, and PPK2B^cg^ from *Corynebacterium glutamicum* was used to catalyze the conversion of ADP into ATP. As a result, the recombinant strain PL05 carrying coexpressed *pap* and *ppk2B*^*cg*^ was constructed, and ε-PL production was 2.34 g/L in shake-flask fermentation, which was an increase of 21.24% compared with *S. albulus* WG608; intracellular ATP was also increased by 71.56%. Although we also tried to develop a dynamic ATP regulation route, the result was not as expected. Finally, the conditions of polyP_6_ addition were optimized for strain PL05 in batch and fed-batch fermentations, and the maximum ε-PL production by fed-batch fermentation in a 5-L fermenter was 59.25 g/L, which is the highest ε-PL production reported by genetically engineered strains. This study provides an efficient approach to improve the production of not only ε-PL but also other ATP-driven metabolites.

## Supplementary Information

**Additional file 1**: **Table S1**. Primers used in this study. **Fig. S1**. Fed-batch fermentation processes of strain PL05 with different polyP_6_ addition strategies. A: *S. albulus* WG608 without polyP_6_ addition. B: strain PL05 without polyP_6_ addition. C: strain PL05 with polyP_6_ addition at the final concentration of 1 g/L every 48 h. D: strain PL05 with polyP_6_ addition at the final concentration of 1 g/L at 0 h and 108 h. The data are presented as averages, and the error bars represent standard deviations (n = 3 independent experiments). **Fig. S2**. Batch-fermentation of* ppk* heterologous expression strains with 1 g/L polyP_6_ addition in 1-L fermenters. The data are presented as averages, and the error bars represent standard deviations (n = 3 independent experiments). * 0.01 < P < 0.05, ** P < 0.01, *** P < 0.001.

## Data Availability

All data generated or analyzed during the current study are included in this manuscript and Additional file 1.

## References

[1] Takeuchi Y, Ushimaru K, Kaneda K, Maruyama C, Ito T, Yamanaka K, Ogasawara Y, Katano H, Kato Y, Dairi T, Hamano Y (2022). First direct evidence for direct cell-membrane penetrations of polycationic homopoly(amino acid)s produced by bacteria. Commun Biol.

[2] Hamano Y, Arai T, Ashiuchi M, Kino K (2013). NRPSs and amide ligases producing homopoly (amino acid)s and homooligo (amino acid)s. Nat Prod Rep.

[3] Tao Y, Chen X, Jia F, Wang S, Xiao C, Cui F, Li Y, Bian Z, Chen X, Wang X (2015). New chemosynthetic route to linear ε-poly-lysine. Chem Sci.

[4] Xu Z, Xu Z, Feng X, Xu D, Liang J, Xu H (2016). Recent advances in the biotechnological production of microbial poly(ɛ-L-lysine) and understanding of its biosynthetic mechanism. Appl Microbiol Biotechnol.

[5] Wang A, Tian W, Cheng L, Xu Y, Yu B (2020). Enhanced ε-poly-L-lysine production by the synergistic effect of ε-poly-L-lysine synthetase overexpression and citrate in *Streptomyces albulus*. Front Bioeng Biotechnol.

[6] Tao YH (2016). New polymerization methodology of amino acid based on lactam polymerization. Acta Polym Sin.

[7] Shima S, Sakai H (1981). Poly-L-lysine produced by *Streptomyces*. part. II taxonomy and fermentation studies. Agric Biol Chem.

[8] Nishikawa M, Ogawa K (2002). Distribution of microbes producing antimicrobial epsilon-poly-L-lysine polymers in soil microflora determined by a novel method. Appl Environ Microbiol.

[9] Ouyang J, Xu H, Li S, Zhu H, Chen W, Zhou J, Wu Q, Xu L, Ouyang P (2006). Production of ε-poly-L-lysine by newly isolated *Kitasatospora* sp PL6-3. J Biotechnol.

[10] Purev E, Kondo T, Takemoto D, Niones JT, Ojika M (2020). Identification of ε-poly-L-lysine as an antimicrobial product from an *epichloë* endophyte and isolation of fungal ε-PL synthetase gene. Molecules.

[11] Bhattacharya S, Dineshkumar R, Dhanarajan G, Sen R, Mishra S (2017). Improvement of ε-poly-lysine production by marine bacterium *Bacillus licheniformis* using artificial neural network modeling and particle swarm optimization technique. Biochem Eng J.

[12] Jiang X, Radko Y, Gren T, Palazzotto E, Lee SY (2021). Distribution of ε-poly-L-lysine synthetases in coryneform bacteria isolated from cheese and human skin. Appl Environ Microbiol.

[13] Wang L, Zhang CY, Zhang JH, Rao ZM, Xu XM, Mao ZG, Chen XS (2021). Epsilon-poly-L-lysine: recent advances in biomanufacturing and applications. Front Bioeng Biotechnol.

[14] Loeffler M, Simen JD, Jaeger G, Schaeferhoff K, Freund A, Takors R (2016). Engineering *E. coli* for large-scale production-strategies considering ATP expenses and transcriptional responses. Metab Eng.

[15] Luo ZS, Zeng WZ, Du GC, Chen J, Zhou J (2019). Enhanced pyruvate production in candida glabrata by engineering ATP futile cycle system. ACS Synth Biol.

[16] Wang WS, Li SS, Li ZL, Zhang JY, Fan KQ, Tan GY, Ai GM, Lam SM, Shui GH, Yang ZH (2020). Harnessing the intracellular triacylglycerols for titer improvement of polyketides in *Streptomyces*. Nat Biotechnol.

[17] Huang R, Liu HL, Zhao WW, Wang SQ, Wang SF, Cai J, Yang C (2022). AdpA, a developmental regulator, promotes epsilon-poly-L-lysine biosynthesis in *Streptomyces albulus*. Microb Cell Fact.

[18] Yamanaka K, Kito N, Imokawa Y, Maruyama C, Utagawa T, Hamano Y (2010). Mechanism of epsilon-poly-L-lysine production and accumulation revealed by identification and analysis of an epsilon-poly-L-lysine-degrading enzyme. Appl Environ Microbiol.

[19] Chen YW, Liao Y, Kong WZ, Wang SH (2020). ATP dynamic regeneration strategy for enhancing co-production of glutathione and s-adenosylmethionine in *Escherichia coli*. Biotechnol Lett.

[20] Wang L, Li S, Zhao J, Liu Y, Chen X, Tang L, Mao Z (2019). Efficiently activated epsilon-poly-L-lysine production by multiple antibiotic-resistance mutations and acidic pH shock optimization in *Streptomyces albulus*. MicrobiologyOpen.

[21] Bankar SB, Singhal RS (2011). Improved poly-epsilon-lysine biosynthesis using *Streptomyces noursei* NRRL 5126 by controlling dissolved oxygen during fermentation. J Microbiol Biotechnol.

[22] Xu Z, Bo F, Xia J, Sun Z, Li S, Feng X, Xu H (2015). Effects of oxygen-vectors on the synthesis of epsilon-poly-lysine and the metabolic characterization of *Streptomyces albulus* PD-1. Biochem Eng J.

[23] Xu Z, Cao C, Sun Z, Li S, Feng X (2015). Construction of a genetic system for *Streptomyces albulus* PD-1 and improving poly(ε-L-lysine) production through expression of *Vitreoscilla* hemoglobin. J Microbiol Biotechnol.

[24] Man Z, Guo J, Zhang Y, Cai Z (2020). Regulation of intracellular ATP supply and its application in industrial biotechnology. Crit Rev Biotechnol.

[25] Andexer JN, Richter M (2015). Emerging enzymes for ATP regeneration in biocatalytic processes. ChemBioChem.

[26] Tavanti M, Hosford J, Lloyd RC, Brown MJ (2021). ATP regeneration by a single polyphosphate kinase powers multigram-scale aldehyde synthesis in vitro. Green Chem.

[27] Lv Q, Hu M, Tian L, Liu F, Wang Q, Xu M, Rao Z (2021). Enhancing L-glutamine production in *Corynebacterium glutamicum* by rational metabolic engineering combined with a two-stage pH control strategy. Bioresour Technol.

[28] Krauser S, Hoffmann T, Heinzle E (2015). Directed multistep biocatalysis for the synthesis of the polyketide oxytetracycline in permeabilized cells of *Escherichia coli*. ACS Catal.

[29] Wu GY, Chen XS, Wang L, Mao ZG (2016). Screening of high-yield ε-poly-L-lysine producing strains through ribosome engineering. Microbiol China.

[30] Wilkinson CJ, Hughes-Thomas ZA, Martin CJ, Bohm I, Mironenko T, Deacon M, Wheatcroft M, Wirtz G, Staunton J, Leadlay PF (2002). Increasing the efficiency of heterologous promoters in *actinomycetes*. J Mol Microbiol Biotechnol.

[31] Pan L, Chen XS, Wang KF, Mao ZG (2019). Understanding high epsilon-poly-L-lysine production by *Streptomyces albulus* using pH shock strategy in the level of transcriptomics. J Ind Microbiol Biotechnol.

[32] Xu R, Wang Y, Huang H, Jin X, Li J, Du G, Kang Z (2021). Closed-loop system driven by ADP phosphorylation from pyrophosphate affords equimolar transformation of ATP to 3’-phosphoadenosine-5’-phosphosulfate. ACS Catal.

[33] Yan XX, Zhang GJ, Bie FJ, Lv YH, Ma Y, Ma M, Wang YR, Hao XQ, Yuan NJ, Jiang XF (2017). Eugenol inhibits oxidative phosphorylation and fatty acid oxidation via downregulation of c-Myc/PGC-1 beta/ERRa signaling pathway in MCF10A-ras cells. Sci Rep.

[34] Pan L, Chen XS, Liu MM, Liu YJ, Mao ZG (2017). Efficient production of epsilon-poly-L-lysine from glucose by two-stage fermentation using pH shock strategy. Process Biochem.

[35] Ren XD, Chen XS, Zeng X, Wang L, Tang L, Mao ZG (2015). Acidic pH shock induced overproduction of epsilon-poly-L-lysine in fed-batch fermentation by *Streptomyces* sp M-Z18 from agro-industrial by-products. Bioprocess Biosyst Eng.

[36] Itzhaki RF (1972). Colorimetric method for estimating polylysine and polyarginine. Anal Biochem.

[37] International A. Official methods of analysis of AOAC international, 16th edition. Volume 2; 1995.

[38] Kimura Y, Kamatani S (2021). Catalytic activity profile of polyP:AMP phosphotransferase from *Myxococcus xanthus*. J Biosci Bioeng.

[39] Chen H, Zhang Y (2020). Enzymatic regeneration and conservation of ATP: challenges and opportunities. Crit Rev Biotechnol.

[40] Huang YJ, Maruyama Y, Dvoryanchikov G, Pereira E, Chaudhari N, Roper SD (2007). The role of pannexin 1 hemichannels in ATP release and cell-cell communication in mouse taste buds. PNAS.

[41] Chivasa S, Ndimba BK, Simon WJ, Lindsey K, Slabas AR (2005). Extracellular ATP functions as an endogenous external metabolite regulating plant cell viability. Plant Cell.

[42] Yamanaka K, Maruyama C, Takagi H, Hamano Y (2008). Epsilon-poly-L-lysine dispersity is controlled by a highly unusual nonribosomal peptide synthetase. Nat Chem Biol.

[43] Meng L, Li M, Yang SH, Kim TJ, Suh JW (2011). Intracellular ATP levels affect secondary metabolite production in *streptomyces* spp. Biosci Biotechnol Biochem.

[44] Winkler HH, Neuhaus HE (1999). Non-mitochondrial ATP transport. Trends Biochem Sci.

[45] Li M, Kim TJ, Kwon HJ, Suh JW (2008). Effects of extracellular ATP on the physiology of *Streptomyces coeficolor* A3(2). FEMS Microbiol Lett.

[46] Kuroda A, Kornberg A (1997). Polyphosphate kinase as a nucleoside diphosphate kinase in *Escherichia coli* and *Pseudomon asaeruginosa*. PNAS.

[47] Kamatani S, Takegawa K, Kimura Y (2018). Catalytic activity profile of polyphosphate kinase 1 from *Myxococcus xanthus*. Curr Microbiol.

[48] Hiraki J, Hatakeyama M, Morita H, Izumi Y (1998). Improved epsilon-poly-L-lysine production of an S-(2-aminoethyl)-L-cysteine resistant mutant of *Streptomyces albulus*. Seibutsu-Kogaku Kais.

[49] Kahar P, Iwata T, Hiraki J, Park EY, Okabe M (2001). Enhancement of epsilon-poly-lysine production by *Streptomyces albulus* strain 410 using pH control. J Biosci Bioeng.

[50] Li S, Li F, Chen XS, Wang L, Xu J, Tang L, Mao ZG (2012). Genome shuffling enhanced epsilon-poly-L-lysine production by improving glucose tolerance of *Streptomyces graminearus*. Appl Biochem Biotechnol.

[51] Wang L, Chen XS, Wu GY, Li S, Zeng X, Ren XD, Tang L, Mao ZG (2015). Improved epsilon-poly-L-lysine production of *Streptomyces* sp. FEEL-1 by atmospheric and room temperature plasma mutagenesis and streptomycin resistance screening. Ann Microbiol.

[52] Zhou YP, Ren XD, Wang L, Chen XS, Mao ZG, Tang L (2015). Enhancement of epsilon-poly-lysine production in epsilon-poly-lysine-tolerant *Streptomyces* sp. by genome shuffling. Bioprocess Biosyst Eng.

[53] Wang L, Chen XS, Wu GY, Zeng X, Ren XD, Li S, Tang L, Mao ZG (2016). Genome shuffling and gentamicin-resistance to improve epsilon-poly-L-lysine productivity of *Streptomyces albulus* W-156. Appl Biochem Biotechnol.

[54] Pan L, Chen XS, Liu MM, Liu YJ, Mao ZG (2017). Efficient production of ε-poly-L-lysine from glucose by two-stage fermentation using pH shock strategy. Process Biochem.

[55] Wang L, Deng Y, Wu MP, Zhang JH, Zhang HJ, Mao ZG, Chen XS (2022). Efficient ε-poly-L-lysine production by *Streptomyces albulus* based on a dynamic pH-regulation strategy. Process Biochem.

[56] Hamano Y, Nicchu I, Shimizu T, Onji Y, Hiraki J, Takagi H (2007). Epsilon-poly-L-lysine producer, *Streptomyces albulus*, has feedback-inhibition resistant aspartokinase. Appl Microbiol Biotechnol.

[57] Gu YY, Wang SF, Geng WT, Wang XM, Yang C, Song CJ (2016). Effects of chromosomal integration of the *Vitreoscilla* hemoglobin gene (*vgb*) and S-adenosylmethionine synthetase gene (*metK*) on epsilon-poly-L-lysine synthesis in *Streptomyces albulus* NK660. Appl Biochem Biotechnol.

[58] Xu D, Yao H, Cao C, Xu Z, Li S, Xu Z, Zhou J, Feng X, Xu H (2018). Enhancement of epsilon-poly-L-lysine production by overexpressing the ammonium transporter gene in *Streptomyces albulus* PD-1. Bioprocess Biosyst Eng.

[59] Xu D, Wang R, Xu Z, Xu Z, Li S, Wang M, Feng X, Xu H (2019). Discovery of a short-chain epsilon-poly-L-lysine and its highly efficient production via synthetase swap strategy. J Agric Food Chem.

[60] Yamanaka K, Hamano Y, Oikawa T (2020). Enhancement of metabolic flux toward epsilon-poly-L-lysine biosynthesis by targeted inactivation of concomitant polyene macrolide biosynthesis in *Streptomyces albulus*. J Biosci Bioeng.

