# Supplementary material for: Engineering Streptomyces albulus to enhance ε-poly-L-lysine production by introducing a polyphosphate kinase-mediated ATP regeneration system
Source: Microb Cell Fact. 2023 Mar 14;22:51. doi: 10.1186/s12934-023-02057-7 (PMC10012588; doi:10.1186/s12934-023-02057-7)
Supplement: Supplementary file 1 — Additional file 1: Table S1. Primers used in this study. Fig. S1. Fed-batch fermentation processes of strain PL05 with different polyP6 addition strategies. A: S. albulus WG608 without polyP6 addition. B: strain PL05 without polyP6 addition. C: strain PL05 with polyP6 addition at the final concentration of 1 g/L every 48 h. D: strain PL05 with polyP6 addition at the final concentration of 1 g/L at 0 h and 108 h. The data are presented as averages, and the error bars represent standard deviations (n = 3 independent experiments). Fig. S2. Batch-fermentation of ppk heterologous expression strains with 1 g/L polyP6 addition in 1-L fermenters. The data are presented as averages, and the error bars represent standard deviations (n = 3 independent experiments). * 0.01 < P < 0.05, ** P < 0.01, *** P < 0.001. [file 12934_2023_2057_MOESM1_ESM.docx]

**Engineering *Streptomyces albulus* to enhance ε-poly-L-lysine production by introducing a polyphosphate kinase-mediated ATP regeneration system**

Hao Yang, Daojun Zhu, Lang Kai, Liang Wang, Hongjian Zhang, Jianhua Zhang, Xusheng Chen^*^

Key Laboratory of Industrial Biotechnology, Ministry of Education; School of Biotechnology, Jiangnan University, Wuxi 214122, China

^*^Correspondence to: Prof. Chen, School of Biotechnology, Jiangnan University, 1800 Lihu Road, Wuxi 214122, Jiangsu, China

Tel & Fax: 0086-510-85918296

E-mail: chenxs@jiangnan.edu.cn

**Table S1** Primers used in this study.

| **Primers** | **Sequence (5’-3’)** | **Description** |
| --- | --- | --- |
| **Construction of *S. albulus* WG608 derivatives** | | |
| *ppk1-F* | ATGCGTGGTCGTCCGCAACC | DNA fragment cloning |
| *ppk1-R* | TCAACGTGGCAGCCGGACTCC |  |
| c-*ppk1*-F | taccgattaaATGCGTGGTCGTCCGCAACC |  |
| c-*ppk1*-R | ctatgacatgattacgaattcTCAACGTGGCAGCCGGACTCC |  |
| c-*ppk2*-F | ggttggtaggatccacatatgATTGGCTAAAATCCACAGCCTT |  |
| c-*ppk2*-R | atccaaagacgcCTAGTCACCGATCTGGTCGCG |  |
| c-*pap*-F-2 | gtgactagGCGTCTTTGGATATTTGTTGTCTTA |  |
| c-*pap*-R-2 | ctatgacatgattacgaattcTTAATCGGTATCTCGATCAGCTTTT |  |
| c-*pap*-F-3 | gtgactagGCGTCTTTGGATATTTGTTGTCTTA |  |
| c-*pap*-R-3 | acgacgaccgTTAATCGGTATCTCGATCAGCTTTT |  |
| pIB-F | CGCCAGGGTTTTCCCAGTCACGAC | Universal primers |
| pIB-R | AGCGGATAACAATTTCACACAGGA |  |
| 2-F | AGTGGCAGAACTGGACCAAGGA | PL05 validation primers |
| 2-R | CTCATCGCATCGGCAACCTGTT |  |
| 3-F | AGTGGCAGAACTGGACCAAGGA | PL06 validation primers |
| 3-R | GATGCTGTGGATGCGGAAGAGG |  |
| **Construction of *E. coli* BL21(DE3) derivatives** | | |
| B-*ppk1*-F | TCCTGGAGCACCACACCTTC | DNA fragment cloning |
| B-*ppk1*-R | ACTTGATGTTGGCGGACTCGT |  |
| B-*ppk2B^cg^*-F | CCTCATCGGTCTGCTTGGCTAA |  |
| B-*ppk2B^cg^*-R | TCGGTGTAACGGAACTGCTCTT |  |
| B-*ppk2C^pa^*-F | CGGCATCTTCTTCGGCAACTGG |  |
| B-*ppk2C^pa^*-R | GCTCCATCTGCGTCTGCTTGTC |  |
| P-F | TGACTGGTGGACAGCAAATGGG | Validation primers |
| P-R | CACTTGATGTTGGCGGACTCGT |  |
| B-F | GCAGCAGCCATCATCATCATCA |  |
| B-R | GCCCACGAAGCACAACCTTAG |  |
| C-F | ATGCGTCCGGCGTAGAGGAT |  |
| C-R | CTCGATGGCGTCGTTGATGGT |  |


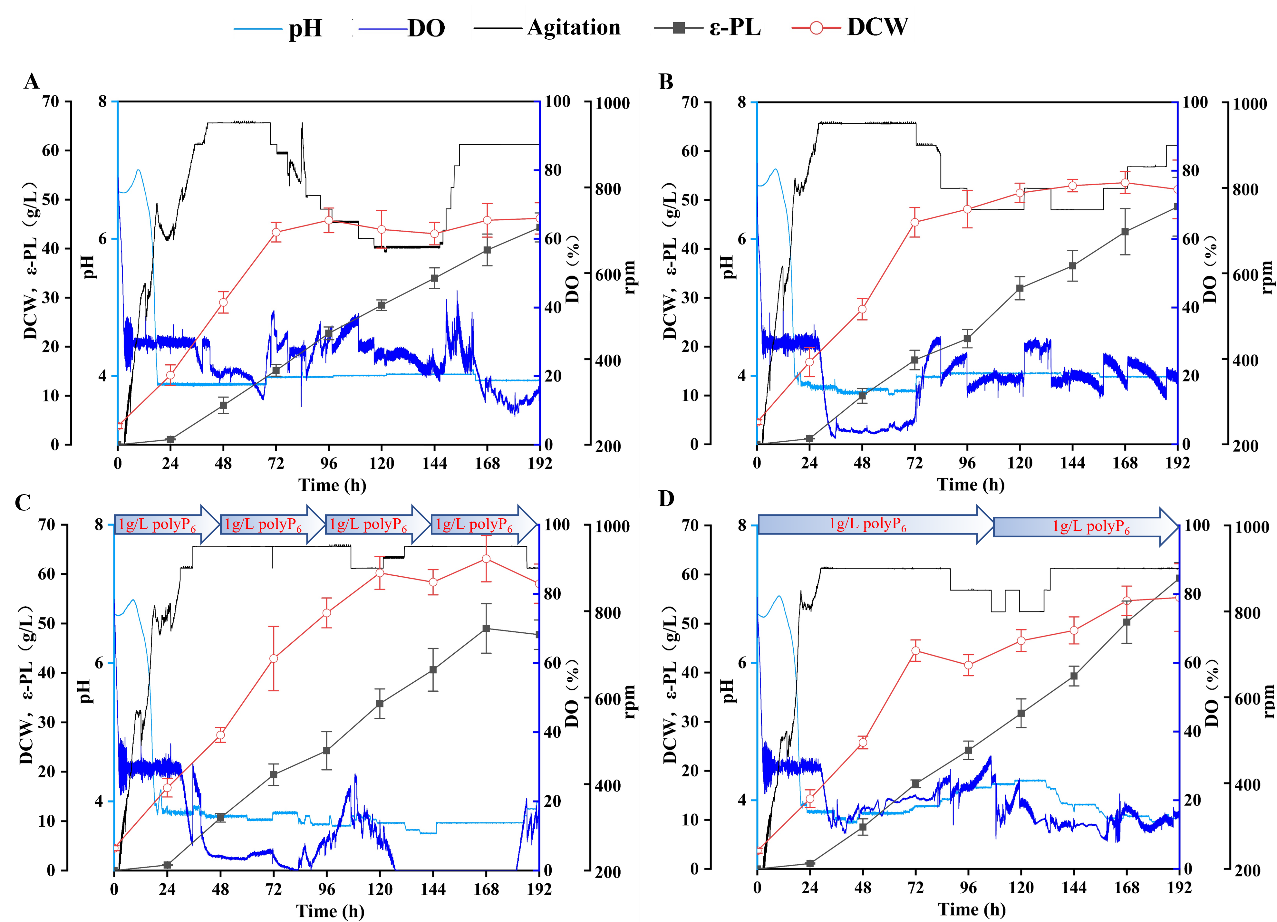


**Fig. S1** Fed-batch fermentation processes of strain PL05 with different polyP_6_ addition strategies. A: *S. albulus* WG608 without polyP_6_ addition. B: strain PL05 without polyP_6_ addition. C: strain PL05 with polyP_6_ addition at the final concentration of 1 g/L every 48 h. D: strain PL05 with polyP_6_ addition at the final concentration of 1 g/L at 0 h and 108 h. The data are presented as averages, and the error bars represent standard deviations (n = 3 independent experiments).


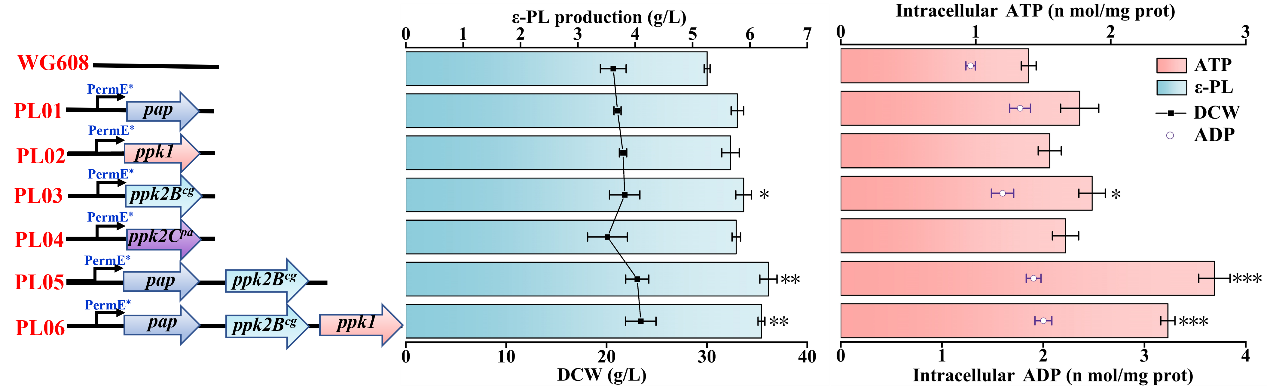


**Fig. S2** Batch-fermentation of *ppk* heterologous expression strains with 1 g/L polyP_6_ addition in 1-L fermenters. The data are presented as averages, and the error bars represent standard deviations (n = 3 independent experiments). * 0.01 < P < 0.05, ** P < 0.01, *** P < 0.001.
